# Redefining meaning and success in science, research and teaching

**DOI:** 10.1038/s44319-024-00116-7

**Published:** 2024-03-20

**Authors:** David R Smith

**Affiliations:** Western Ontario University, London, ON Canada

**Keywords:** Careers, History & Philosophy of Science

## Abstract

The mid-life crisis to find meaning in life affects even academics. At the end of the day, however you redefine your goals in life, it is interactions with fellow humans and maintaining a positive spirit that keeps us moving on.

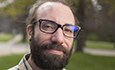

Plato says that the unexamined life is not worth living. But what if the examined life turns out to be a clunker as well?

Kurt Vonnegut

I recently returned to work after an extended sick leave. It’s funny the things I missed when I was away, like my wobbly, antique-oak desk chair, the overpriced campus coffee, saranwrap-infused sandwiches, and the pitapat of undergraduate footsteps outside my office. Most of all, I missed informal conversations with students and colleagues. I used to be dismissive of workplace banter, believing I had more important things to be doing. But my time away has taught me that you can learn a great deal and sometimes help a whole lot by listening to other people’s stories and problems. So at least once or twice a week, I keep my office door propped open, which as we all know is the universal sign for “Please come in and disturb me.” In the past 3 months, these invited disruptions have given me new insights into my workplace and the people in it. And although less productive, I am a wiser and more content person for having had them.

Last week I had a persistent giggle after a fellow professor recounted to me the harrowing rescue of an iPhone. She had accidentally and unknowingly dropped her phone into the toilet during a lecture intermission. When class resumed, a student politely let it be known that they had just spotted in the washroom an unsuspecting phone submerged in toilet water, which thankfully, they added, was still receiving notifications. Of course, everyone quickly checked their pockets and backpacks, the instructor included, who then reflexively mouthed the words “Shit!” after which someone in the front row replied, “Let us hope for your sake, professor, that it is just pee.” Ultimately, everyone involved was happy to learn that the current-generation iPhone is a resilient piece of engineering.

But I do not always come away from these conversations with a smile. The other day an upper-year undergraduate stopped by to ask how I was faring after my illness. They say that the fruit of suffering is empathy, and for this young person this had already proven true. After listening attentively to me recount my own problems, she described how her mother had recently passed away from breast cancer. “It’s strange,” she said, “you would think that after all this I would be even more motivated to get into medical school, maybe even become an oncologist, but all I want to do is sit in a chair and look out the window or spend time with friends and family. I’m no longer interested in becoming a doctor or scientist.” “Be patient and kind to yourself,” I said. “Things may shift in a few months or years.” As this brave, solicitous individual said her goodbyes, I appreciated more than ever that so many among us are navigating the most challenging of circumstances.

If I had to distill into one sentence the overarching sentiment that has come from my chats with staff and students, it would be something like “the enduring struggle to find meaning in life, work and relationships.” There are those lucky few who find a niche early in their scientific journey and remain content throughout their careers, provided they have lab space, some grant money and time to tinker away at research. I would argue, however, that this is not the case for most of us. Whether you are a student, postdoc or faculty member, there have likely been multiple instances when you have felt directionless, unsatisfied or uninterested in your work. Sometimes all it takes is a little time and perseverance to get out of a rut. But at other times, it can require more significant changes, such as re-examining and redefining what brings value and purpose to your work.

In my department, I know a few people who were highly productive and successful researchers but midway through their careers shifted their focus to undergraduate teaching and are now equally successful at that. Other colleagues, however, still roll their eyes at this decision, noting that “it is such a shame that so-and-so gave up lab work.” We have all heard similar criticisms levied against those who transition from research into administration or from academia into the private sector. In my experience, those who are most critical of others are often the first to complain about their own lot at the university and would benefit from some self-reflection. It is unlikely that a single subject or goal will sustain you throughout your entire career. It is better to pivot towards something that reinvigorates you, even if it is less prestigious or “important”, than to become bitter and resentful.

Do not get me wrong, I have yet to have someone say to me, “Dave, I am so inspired and satisfied by my work I could give you a platonic, consensual hug!” But there are certainly people I interact with who exude quiet enthusiasm and tenacity for what they are doing, and then there are those who do not. I am currently wrestling with these ideas myself, trying to shift my attitude toward the positive and redefine my goals with intention. I’ve spent years studying and writing about the genomes within mitochondria and chloroplasts. Some of this research has been impactful and some has not. But I wonder: Does the world need another organelle genome paper from David Smith? Do I even want to write another one? Well, you might be thinking, it is certainly better than this self-help blither blabber you are pumping out. But the challenge for me is the same as it is for you: to strive to find meaning and satisfaction in daily actions and have a constructive impact on students and colleagues. Tomorrow, I may find purpose in deciphering the mitochondrial DNA of a little-known unicellular eukaryote, but today I’m finding it by reaching out through these words and through an office door that is propped open just enough to invite people in to share their stories.

Earlier today, an old, silverback professor emeritus poked his head through the crack in the door and said in a loud, happy voice: “Young man, how are you keeping on?” Not taking my own advice, I moaned and whined a bit about work and various global catastrophes. He smiled and said, “Dave, I recommend you revisit the famous quote by Voltaire: ‘The most important decision you make is to be in a good mood.’” Then he turned away and kept bopping down the hallway, like a first-year undergraduate on the way to an exciting lecture.

